# Incidence, Correlates, and Prognostic Implications of New-Onset Atrial Fibrillation in Adults With Repaired Coarctation of Aorta

**DOI:** 10.1016/j.cjcpc.2024.07.004

**Published:** 2024-08-08

**Authors:** Alexander C. Egbe, Malini Madhavan, Heidi M. Connolly, Ahmed E. Ali, Ahmed Younis, Abhishek Deshmukh

**Affiliations:** Department of Cardiovascular Medicine, Mayo Clinic, Rochester, Minnesota, USA

## Abstract

**Background:**

There are limited data about the incidence and outcomes of atrial fibrillation (AF) in adults with coarctation of aorta (COA). The purpose of this study was to determine the incidence, correlates, and prognostic implications of new-onset AF in adults with repaired COA.

**Methods:**

A retrospective cohort study of adults with repaired COA without a prior history of atrial arrhythmias was performed. We reviewed rhythm data (electrocardiogram, Holter, and rhythm strip) obtained from baseline to the last clinical encounter. The correlates of AF and the relationship between AF and cardiovascular adverse events (heart failure hospitalization and/or all-cause mortality) were assessed using Cox regression.

**Results:**

Of 782 patients (aged 32 [interquartile range: 21-43] years; 462 [59%] men), 42 (5.4%) developed new-onset AF. The incidence of new-onset AF was 9 per 1000 patient-years (0.9% per year), and the median age at onset of AF was 36 (interquartile range: 24-49) years. The correlates of new-onset AF were older age, hypertension, left atrial dysfunction, and left ventricular hypertrophy. Of 782 patients, 92 (12%) had cardiovascular adverse events. On multivariable analysis, new-onset AF was associated with cardiovascular adverse events (hazard ratio: 1.09, 95% confidence interval: 1.03-1.15), after adjustment for age, hypertension, and right and left ventricular structure and function.

**Conclusions:**

Patients with COA were at risk for developing AF at a relatively young age (median age: 36 years), and AF was associated with cardiovascular adverse outcomes. There is a need to target the modifiable risk factors for AF to reduce the adverse outcomes associated with AF.

## Graphical abstract

**Figure:**
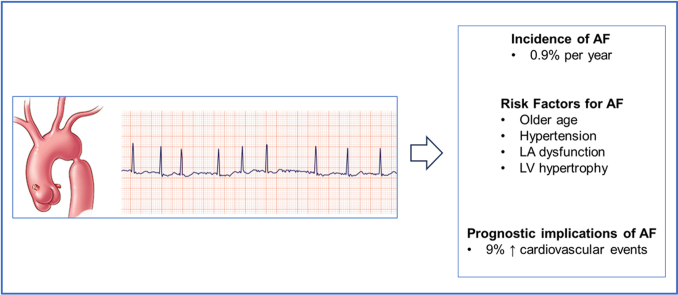

## Résumé

**Contexte:**

Les données relatives à l’incidence et à l’issue de la fibrillation auriculaire chez les adultes présentant une coarctation de l’aorte sont limitées. Le but de cette étude était de déterminer l’incidence, les corrélats et la signification pronostique d’une fibrillation auriculaire d’apparition nouvelle chez des adultes présentant une coarctation de l’aorte réparée.

**Méthodes:**

Étude de cohorte rétrospective menée auprès d’adultes présentant une coarctation de l’aorte réparée en l’absence d’antécédents d’arythmie auriculaire. Nous avons examiné les données relatives au rythme (électrocardiogramme, Holter et bande de rythme) obtenues entre le début de l’étude et la dernière consultation en clinique. Les corrélats de la fibrillation auriculaire et la relation entre la fibrillation auriculaire et les événements cardiovasculaires indésirables (hospitalisation pour insuffisance cardiaque et/ou mortalité toutes causes confondues) ont été évalués à l’aide d’un modèle de régression de Cox.

**Résultats:**

Parmi les 782 patients (âge : 32 ans [intervalle interquartile : 21 à 43]; 462 hommes [59 %]), 42 (5,4 %) ont présenté une fibrillation auriculaire d’apparition nouvelle. L’incidence de la fibrillation auriculaire d’apparition nouvelle était de 9 cas pour 1 000 années-patients (0,9 %/an), et l’âge médian au moment de l’apparition de la fibrillation auriculaire était de 36 ans (intervalle interquartile : 24 à 49). Les corrélats de la fibrillation auriculaire d’apparition nouvelle ont été l’âge avancé, l’hypertension, la dysfonction auriculaire gauche et l’hypertrophie ventriculaire gauche. Parmi les 782 patients, 92 (12 %) ont présenté des événements cardiovasculaires indésirables. D’après une analyse multivariée, la fibrillation auriculaire d’apparition nouvelle était associée aux événements cardiovasculaires indésirables (rapport des risques instantanés : 1,09; intervalle de confiance à 95 % : 1,03 à 1,15) après correction tenant compte de l’âge, de l’hypertension ainsi que de la structure et de la fonction ventriculaire gauche et droite.

**Conclusions:**

Les patients présentant une coarctation de l’aorte étaient exposés à un risque de fibrillation auriculaire à un âge relativement précoce (âge médian : 36 ans), et la fibrillation auriculaire a été associée à certains événements cardiovasculaires indésirables. Il est nécessaire de cibler les facteurs de risque modifiables de fibrillation auriculaire pour réduire les issues indésirables associées à celle-ci.

Atrial fibrillation (AF) is now the most common atrial arrhythmia in adults with congenital heart disease (CHD) over the age of 50 years.^1^^,^^2^ The prevalence of AF differs across CHD diagnoses and tends to be higher in patients with left-sided lesions.^1^^,^^2^ Coarctation of aorta (COA) is characterized by chronic left ventricular (LV) pressure overload, leading to increased cardiomyocyte hypertrophy and fibrosis, and in turn, increased myocardial stiffness.^3^, ^4^, ^5^ These structural and functional changes lead to high left atrial (LA) pressures and LA remodelling, thus providing critical substrates for the initiation and propagation of AF.^4^^,^^6^^,^^7^

Although patients with COA have all the prerequisite substrates for AF, there are limited data about the incidence and prognostic implications of AF in this population. The purpose of this study was to determine the incidence, correlates, and prognostic implications of new-onset AF in adults with repaired COA.

## Methods

### Study population

This is a retrospective cohort study of adults (age ≥18 years) with repaired COA who had more than 12 months of follow-up at Mayo Clinic between January 1, 2003, and December 31, 2022. From this cohort, we excluded patients with a prior history of atrial arrhythmias (defined as AF, atrial flutter, or atrial tachycardia) and patients with Shone’s complex (COA with LV inflow disease).^8^ We defined COA with concomitant LV outflow tract (LVOT) disease as the presence of ≥moderate LVOT stenosis (LVOT Doppler mean gradient >20 mm Hg) and/or ≥moderate aortic regurgitation.^8^ Patients with COA without concomitant LVOT disease were considered to have isolated COA.

### Data acquisition

The first clinical encounter within the study period was considered the baseline encounter for this study, and the clinical indices obtained within 6 months from the baseline encounter were used to define the baseline characteristics of the patients.

#### Rhythm monitoring

AF was defined as irregular tachyarrhythmia lasting longer than 30 seconds and characterized by lack of consistent atrial activity/p-wave on electrocardiogram, Holter monitor, or rhythm strip. AF was classified as paroxysmal AF if it terminated spontaneously or by chemical/electrical cardioversion within 7 days from onset, or persistent AF if it lasts for more than 7 days.^1^^,^^2^^,^^9^ Episodes of postoperative AF, defined as AF occurring within 30 days from the time of surgery, were excluded because these were considered as provoked events.

#### Echocardiography

Cardiac structure and function were assessed by 2-dimensional, Doppler, and speckle tracking echocardiography using a standard technique.^10^ The procedural details for performing comprehensive echocardiography in our laboratory have been described.^8^^,^^11^ Chamber function (right atrium, right ventricle [RV], LA, and LV) was assessed using strain imaging. Chamber size and haemodynamics were assessed using 2-dimensional and Doppler echocardiography.

#### Cardiovascular outcomes

The prognostic implications of AF were determined by assessing the relationship between new-onset AF and cardiovascular adverse events defined as the composite outcome of heart failure hospitalization and/or all-cause mortality. Exploratory analysis was performed to assess the relationship between new-onset AF, heart failure hospitalization, and all-cause mortality, as separate outcomes. The occurrence of cardiovascular adverse events was ascertained as time-to-event endpoint from baseline encounter to the occurrence of events. The patients without cardiovascular events were censored at the last clinical encounter or December 31, 2022.

### Statistical analysis

Data were presented as mean ± standard deviation, median (interquartile range), and count (%). New-onset AF was assessed as a time-to-event outcome, and the incidence of new-onset AF was expressed as events per 1000 patient-years as well as % per year. The correlates of new-onset AF were determined using multivariable Cox regression analysis. First, we performed univariable analysis to assess the relationship between individual covariates (demographic indices, anatomic/surgical indices, comorbidities, and echocardiographic indices of right and left heart structure and function) and occurrence of new-onset AF. These variables were chosen based on clinical knowledge and relevance. The covariates with *P* < 0.1 on univariable analysis were then entered into the multivariable model, and the final covariate selection was based on stepwise backward variable selection with a *P* value of <0.1 required for a covariate to remain in the model. Similarly, the relationship between new-onset AF and cardiovascular outcomes was assessed using multivariable Cox regression analysis, and new-onset AF was modelled as time-dependent covariate. The single conditional imputation method was used to correct for missing data.^12^ All statistical analyses were performed with BlueSky Statistics software (version 7.10; BlueSky Statistics LLC, Chicago, IL) and JMP software (version 17.1; SAS Institute Inc, Cary, NC); a *P* value of <0.05 was considered statistically significant.

## Results

### Baseline characteristics

There were 782 patients with repaired COA who met the study inclusion criteria. Table 1 shows the baseline clinical characteristics of the cohort. The median age at baseline encounter was 32 (interquartile range: 21-43) years, and 462 (59%) were men. The median age at the time of initial COA repair was 3 (0.4-16) years, and the COA repair techniques were end-to-end anastomosis (289, 32%), patch aortoplasty (134, 15%), interposition graft (159, 18%), subclavian flap repair (87, 10%), extra-anatomic graft repair (62, 7%), and transcatheter COA stent repair (51, 6%).

**Table 1:** Baseline characteristics (N = 782)

Characteristic	Value
Age (y), median (IQR)	32 (21-43)
Male, n (%)	462 (59)
Body mass index (kg/m^2^), mean ± SD	26 ± 5
Anatomic/surgical history, n (%)	
Bicuspid aortic valve	471 (60)
Isolated COA	641 (82)
Concomitant LVOT disease	141 (18)
Comorbidities, n (%)	
Hypertension	422 (54)
Coronary artery disease	49 (6)
Diabetes	44 (7)
Medications, n (%)	
Diuretics	96 (13)
β-Blockers	218 (28)
ACEI/ARB	306 (39)
MRA	71 (9)
Calcium channel blockers	96 (11)
Laboratory data	
Haemoglobin (g/dL), mean ± SD	13.5 ± 2.1
GFR (mL/min/1.73 m^2^), mean ± SD	98 ± 22
NT-proBNP (pg/mL), median (IQR)	136 (51-307)

Concomitant LVOT disease was defined as having any of the following conditions: aortic valve prosthesis, subvalvular, valvular, or supravalvular aortic stenosis (mean gradient >20 mm Hg), or ≥moderate aortic regurgitation. Isolated COA was defined as COA without associated LVOT disease.

ACEI/ARB, angiotensin-converting enzyme inhibitor/aldosterone receptor blocker; COA, coarctation of aorta; GFR, glomerular filtration rate; LVOT, left ventricular outflow tract; MRA, mineralocorticoid receptor antagonist; NT-proBNP, N-terminal prohormone brain natriuretic peptide.

Table 2 shows the baseline echocardiographic indices of the cohort. The average COA Doppler mean gradient was 13 (9-19) mm Hg, LA reservoir strain was 37% ± 8%, LV global longitudinal strain was –22% ± 5%, right atrium reservoir strain was 39% ± 9%, and RV free wall strain was –27% ± 11%. Of the 782 patients, 141 (18%) had concomitant LVOT disease defined as ≥moderate LVOT stenosis (LVOT Doppler mean gradient >20 mm Hg) and/or ≥moderate aortic regurgitation. Of the 141 patients with LVOT disease, 102 (72%) had isolated LVOT stenosis, 7 (5%) had isolated aortic regurgitation, whereas 32 (23%) had mixed lesion.

**Table 2:** Echocardiography (N = 782)

Variable	Value
Left heart indices	
LA reservoir strain (%)	37 ± 8
LA volume index (mL/m^2^)	28 ± 6
Mitral E velocity (m/s)	1.1 ± 0.4
Septal e′ velocity (cm/s)	9 ± 3
Lateral e′ velocity (cm/s)	13 ± 3
Septal E/e	11 ± 4
Lateral E/e	8 ± 2
LV end-diastolic volume index (mL/m^2^)	58 ± 12
LV end-systolic volume index (mL/m^2^)	20 ± 9
LV ejection fraction (%)	64 ± 7
LV global longitudinal strain (%)	–22 ± 5
LV mass index (g/m^2^)	91 ± 18
Aortic valve peak velocity (m/s)	1.8 (1.5-2.5)
COA mean gradient (mm Hg)	13 (9-19)
Right heart indices	
RA reservoir strain (%)	39 ± 11
RA volume index (mL/m^2^)	24 ± 9
RA pressure (mm Hg)	6 ± 2
RV systolic pressure (mm Hg)	36 ± 9
RV free wall strain (%)	–27 ± 11

Data are presented as mean ± standard deviation and median (interquartile range).

COA, coarctation of aorta; E, mitral early diastolic velocity; e′, mitral annular tissue Doppler early velocity; LA, left atrium; LV, left ventricle; RA, right atrium; RV, right ventricle.

### New-onset AF

The 782 patients were followed for 6.2 (3.9-8.7) years, and during this period, 3017 electrocardiograms (average of 4 per patient) and 216 Holter monitors (average of 0.3 per patient) were performed. Of the 782 patients, 42 (5.4%) developed new-onset AF (all paroxysmal AF) during a total follow-up of 4831 patient-years. The incidence of new-onset AF was 9 per 1000 patient-years (0.9% per year), and the median age at onset of AF was 36 (24-49) years. The median interval between initial COA repair and new-onset AF was 26 (22-30) years.

Of the 42 patients, 26 (62%) underwent direct current cardioversion, followed by initiation/uptitration of class 2 antiarrhythmic drug therapy (n = 18), class 3 antiarrhythmic drug therapy (n = 3), and class 4 antiarrhythmic drug therapy (n = 5). Of the 42 patients, 16 (38%) has spontaneous conversion to sinus rhythm, and this was followed by initiation/uptitration of class 2 antiarrhythmic drug therapy (n = 13) and class 4 antiarrhythmic drug therapy (n = 3). Seven of the 42 (17%) patients subsequently underwent catheter ablation because of recurrent paroxysmal AF. The average CHA_2_DS_2_-VASc (**C**ongestive Heart Failure, **H**ypertension, **A**ge [≥75 years], **D**iabetes, **S**troke/Transient Ischemic Attack, **V**ascular Disease, **A**ge [65-74 years], **S**ex [Female]) scores were as follows: 0 (n = 17), 1 (n = 20), and 2 (n = 5). Of the 42 patients, 12 (29%) received vitamin K antagonist, 17 (41%) received direct oral anticoagulants, and 13 (31%) were on antiplatelet therapy only.

Table 3 shows the univariable models of the correlates of new-onset AF. On multivariable analysis, older age (hazard ratio [HR]: 1.03, 95% confidence interval [CI]: 1.01-1.05, *P* < 0.001), hypertension (HR: 1.48, 95% CI: 1.17-1.83, *P* = 0.02), LA reservoir strain (HR: 0.97, 95% CI: 0.96-0.98, *P* < 0.001), and LV mass index (HR: 1.04, 95% CI: 1.01-1.07, *P* = 0.03) were associated new-onset AF (Table 4) (see Graphical abstract).

**Table 3:** Univariable Cox regression model assessing the correlates of new-onset atrial fibrillation

Characteristic	HR (95% CI)	*P*
Age (y)	1.06 (1.04-1.08)	<0.001
Male	0.91 (0.58-1.40)	0.7
Body mass index (kg/m^2^)	1.03 (0.97-1.09)	0.4
Age of COA repair, (y)	1.02 (0.91-1.12)	0.5
Concomitant LVOT disease	1.18 (0.94-1.42)	0.7
Comorbidities		
Hypertension	2.21 (1.41-3.58)	<0.001
Coronary artery disease	2.40 (1.16-4.43)	0.009
Diabetes	1.09 (0.97-1.20)	0.1
Left heart indices		
LA reservoir strain (%)	0.95 (0.93-0.98)	<0.001
LA volume index (mL/m^2^)	1.05 (1.03-1.06)	<0.001
Septal E/e	1.07 (1.04-1.11)	<0.001
LV global longitudinal strain (%)	0.91 (0.88-0.95)	<0.001
LV mass index (g/m^2^)	1.08 (1.04-1.12)	0.008
Aortic valve peak velocity (m/s)	1.23 (0.90-1.53)	0.4
COA mean gradient (mm Hg)	1.09 (0.94-1.26)	0.5
Right heart indices		
RA reservoir strain (%)	0.97 (0.95-0.99)	0.003
RA volume index (mL/m^2^)	1.03 (1.01-1.05)	0.005
RA pressure (mm Hg)	1.12 (1.04-1.21)	0.002
RV systolic pressure (mm Hg)	1.03 (1.01-1.05)	0.004
≥Moderate tricuspid regurgitation	1.22 (0.79-3.89)	0.7
RV free wall strain (%)	0.95 (0.91-0.99)	0.03

CI, confidence interval; COA, coarctation of aorta; E, mitral early diastolic velocity; e′, mitral annular tissue Doppler early velocity; HR, hazard ratio; LA, left atrium; LVOT, left ventricular outflow tract; RA, right atrium; RV, right ventricle.

**Table 4:** Multivariable Cox regression model assessing the correlates of new-onset atrial fibrillation

Characteristic	HR (95% CI)	*P*
Age (y)	1.03 (1.01-1.05)	<0.001
Hypertension	1.48 (1.17-1.83)	0.02
LA reservoir strain (%)	0.97 (0.96-0.98)	<0.001
LV mass index (g/m^2^)	1.04 (1.01-1.07)	0.03

CI, confidence interval; HR, hazard ratio; LA, left atrium; LV, left ventricle.

### Cardiovascular outcomes

Of the 782 patients, 46 (5.9%) were hospitalized for heart failure and 77 (10%) died during follow-up. The composite cardiovascular adverse event endpoint (heart failure hospitalization and/or all-cause mortality) occurred in 92 (12%) patients. Table 5 shows the univariable models of the correlates of cardiovascular outcomes. On multivariable analysis, new-onset AF was associated with cardiovascular adverse events (HR: 1.09, 95% CI: 1.03-1.15, *P* = 0.02), after adjustment for age (HR: 1.03, 95% CI: 1.01-1.05, *P* < 0.001), hypertension (HR: 1.24, 95% CI: 1.10-1.45, *P* = 0.03), RV systolic pressure (HR: 1.07, 95% CI: 1.02-1.13, *P* = 0.03), RV free wall strain (HR: 0.96, 95% CI: 0.94-0.98, *P* = 0.01), and LV mass index (HR: 1.03, 95% CI: 1.01-1.05, *P* = 0.04) (Table 6). Exploratory analysis showed that new-onset AF was also associated with heart failure hospitalization (HR: 1.13, 95% CI: 1.07-1.19, *P* = 0.007) and all-cause mortality (HR: 1.06, 95% CI: 1.02-1.10, *P* = 0.04) when these outcomes were analysed separately.

**Table 5:** Univariable Cox regression model assessing the correlates of cardiovascular outcomes

Characteristic	HR (95% CI)	*P*
Age (y)	1.04 (1.02-1.06)	<0.001
Male	0.94 (0.88-0.99)	0.03
Body mass index (kg/m^2^)	1.02 (0.95-1.09)	0.5
Age of COA repair (y)	1.01 (0.92-1.10)	0.4
Concomitant LVOT disease	1.09 (1.01-1.17)	0.02
Comorbidities		
Hypertension	1.28 (1.19-2.64)	0.02
Coronary artery disease	1.53 (1.12-3.05)	0.01
Diabetes	1.06 (0.94-1.14)	0.6
Left heart indices		
LA reservoir strain (%)	0.95 (0.93-0.97)	<0.001
LA volume index (mL/m^2^)	1.09 (1.04-1.14)	<0.001
Septal E/e	1.04 (0.96-1.10)	0.2
LV global longitudinal strain (%)	0.94 (0.90-0.98)	0.007
LV mass index (g/m^2^)	1.06 (1.02-1.09)	0.01
Aortic valve peak velocity (m/s)	1.18 (0.93-1.46)	0.2
COA mean gradient (mm Hg)	1.05 (0.92-1.23)	0.4
Right heart indices		
RA reservoir strain (%)	0.95 (0.93-0.98)	<0.001
RA volume index (mL/m^2^)	1.04 (1.02-1.06)	0.002
RA pressure (mm Hg)	1.10 (1.05-1.15)	0.008
RV systolic pressure (mm Hg)	1.04 (1.02-1.06)	<0.001
≥Moderate tricuspid regurgitation	1.17 (0.19-3.89)	0.6
RV free wall strain (%)	0.96 (0.94-0.98)	0.02
Laboratory data		
GFR (mL/min/1.73 m^2^)	0.96 (0.93-0.99)	0.02
NT-proBNP (pg/mL)	1.33 (1.06-1.67)	0.01

RV and LV strain were modelled as absolute values without the negative sign.

CI, confidence interval; COA, coarctation of aorta; E, mitral early diastolic velocity; e’, mitral annular tissue Doppler early velocity; GFR, glomerular filtration rate; HR, hazard ratio; LA, left atrium; LVOT, left ventricular outflow tract; NT-proBNP, N-terminal prohormone brain natriuretic peptide; RA, right atrium; RV, right ventricle.

**Table 6:** Multivariable Cox regression model assessing the correlates of cardiovascular outcomes

Characteristic	HR (95% CI)	*P*
Age (y)	1.03 (1.01-1.05)	<0.001
Hypertension	1.24 (1.10-1.45)	0.03
RV systolic pressure (mm Hg)	1.07 (1.02-1.13)	0.03
RV free wall strain (%)	0.96 (0.94-0.98)	0.01
LV mass index (g/m^2^)	1.03 (1.01-1.05)	0.04
New-onset AF	1.09 (1.03-1.15)	0.02

RV and LV strain were modelled as absolute values without the negative sign. New-onset AF was modelled as time-dependent covariate.

AF, atrial fibrillation; CI, confidence interval; HR, hazard ratio; RV, right ventricle.

## Discussion

The current study assessed the incidence, correlates, and prognostic implications of new-onset AF in adults with repaired COA. The main findings are as follows: (1) the annual incidence of new-onset AF was 9 per 1000 patient-years (0.9% per year), and the correlates of new-onset AF were older age, hypertension, LA reservoir strain, and LV mass index; and (2) new-onset AF was associated with heart failure hospitalization and all-cause mortality.

Atrial arrhythmias are relatively common in adults with CHD and are associated with clinical outcomes.^1^^,^^2^ In a multicentre study of 482 adults with CHD who had documented atrial arrhythmias, Labombarda et al.^1^ reported that, although atrial flutter was the predominant presenting arrhythmia (62% of arrhythmias in the cohort), AF was the predominant presenting arrhythmia in older patients (52% of arrhythmias in patients above 50 years of age). In a different study, Teuwen et al.^2^ analysed data from 199 patients with CHD and documented AF and observed that the age at the time of initial diagnosis of AF was 49 years, and this was significantly less than the age of onset of AF in patients with acquired heart disease.^2^^,^^7^^,^^13^, ^14^, ^15^, ^16^ This suggests that the atrial remodelling from the haemodynamic impact of underlying structural heart disease, as well as myocardial injury from prior cardiac surgeries, likely contributed to the propensity to develop AF at a much younger age in patients with CHD. Although the above studies highlighted the importance of AF in adults with CHD, both studies were based exclusively on cohorts of patients with atrial arrhythmias and hence do not provide data regarding disease incidence and prevalence. In addition, patients with COA were under-represented in both studies, thus limiting the generalizability of these data to the COA population.^1^^,^^2^ The current study addressed these knowledge gaps, by providing estimates of the incidence and correlates of new-onset AF, as well as the prognostic implications of AF in patients with COA. The incidence of new-onset AF observed in the current study is consistent with previous estimates of new-onset AF after cardiac surgery previously reported in our cohort.^17^ Of 1291 (81%) adults with CHD without a prior history of atrial arrhythmias who underwent cardiac surgery, the annual incidence of new-onset AF was 1.5% per year, and the 5-year cumulative incidence of new-onset AF was 8%.^17^ We postulate that the slightly higher incidence of AF in the post–cardiac surgery cohort may be related to myocardiac injury and stress of surgery.^17^ Other studies have reported a baseline prevalence of AF of approximately 10%-15%.^18^

In a prior study, Labombarda et al.^7^ reported an association between LA function and atrial arrhythmias based on a cross-sectional analysis of 56 patients with COA. This is consistent with the results of the current study identifying LA function as a correlate of new-onset AF and also consistent with the robust data from the acquired heart disease population highlighting the central role of LA remodelling in the pathogenesis of AF.^7^^,^^13^, ^14^, ^15^, ^16^ We postulate that the younger age at the time of AF onset observed in the current study may be related to advanced LA remodelling due to chronic exposure to high LV filling pressures.^3^, ^4^, ^5^ The associations between LV afterload, LV remodelling, and LA dysfunction in adults with COA have been described in previous studies.^3^, ^4^, ^5^

Considering the negative prognostic implications of AF (association with heart failure hospitalization and all-cause mortality) observed in this study, there is a need for interventions to modify the risk factors associated with new-onset AF. This is an important initial step towards improving the long-term survival of adults with COA. Of the correlates of AF observed in this study, hypertension is a modifiable risk factor and therefore provides a viable therapeutic target for intervention.^19^, ^20^, ^21^ Such clinical interventions would include multimodality screening for hypertension, intensive medical therapy for hypertension, and early detection and treatment of residual/recurrent COA.^19^, ^20^, ^21^, ^22^ There is also a need to identify and treat other causes of LV pressure overload such as LVOT disease.^23^^,^^24^ There is also a need to determine the optimal rhythm monitoring and clinical surveillance strategy to improve early diagnoses of AF in this population. Although the annual incidence of new-onset AF of 1% per year may not be high enough to warrant routine use of Holter monitoring, perhaps the risk factors for new-onset AF, proposed in this study, can be used to identify patients with a higher likelihood of developing new-onset AF and then implementing rhythm monitoring in such patients. Such risk factor should include older age, diagnosis of hypertension, and presence of LV hypertrophy. Further studies are required to determine the optimal cutoff point for these risk factors to implement rhythm monitoring.

### Limitations

This is a retrospective, single-centre study, and it is therefore prone to selection and ascertainment bias. There was no standardized protocol for rhythm monitoring, and therefore, we could have underestimated the incidence of AF. The study design did not allow for the assessment of the relative efficacy of the different antiarrhythmic therapies. The median COA Doppler mean gradient was only 13 mm Hg, and hence, the lack of association between residual COA Doppler mean gradient and new-onset AF may be related to the low proportion of patients with haemodynamically significant COA.

## Conclusions

Patients with COA were at risk for developing AF at a relatively young age (median age 36 years), and AF was associated with cardiovascular outcomes such as heart failure hospitalization and all-cause mortality. There is a need to target the modifiable risk factors for new-onset AF as an initial step towards improving the long-term survival of adults with COA. Further studies are required to determine whether such interventions would reduce the incidence of new-onset AF and improve outcomes in this population.
